# Aversive Reaction Between Disulfiram and Betel Quid Among Inpatients With Alcohol Use Disorder in Bhutan: A Preliminary Study

**DOI:** 10.1177/10105395231204801

**Published:** 2023-10-14

**Authors:** Ugyen Dem, Zimba Letho, Chencho Dorji, Damber K. Nirola, Sonam Choki, Tashi Dorji, Pelden Chejor

**Affiliations:** 1 Department of Psychiatry, Jigme Dorji Wangchuck National Referral Hospital, Thimphu, Bhutan; 2 Faculty of Nursing and Public Health, Khesar Gyalpo University of Medical Sciences of Bhutan, Thimphu, Bhutan; 3 Centre for Research in Aged Care, Edith Cowan University, Joondalup, Western Australia, Australia

**Keywords:** alcohol use disorders, aversive reaction, betel quid, Bhutan, disulfiram

## Abstract

Betel quid (BQ) is commonly used in the Asia-Pacific region. Disulfiram is prescribed for people with alcohol use disorders (PwAUDs) after the completion of detoxification as an alternative to rehabilitation. This prospective observational study reported the aversive reactions and common symptoms of disulfiram and BQ in PwAUDs. Participants included PwAUDs admitted to the psychiatric ward at the Jigme Dorji Wangchuck National Referral Hospital for detoxification, who were on Disulfiram and using BQ at the same time. Aversive reactions between disulfiram and BQ were observed for 100 patients over a year. Twenty participants showed aversive reactions between BQ and disulfiram. Common symptoms included sweating, diarrhea, dizziness, tremors, palpitations, shortness of breath, nausea and vomiting, and headache. Since PwAUDs in Bhutan are inducted on disulfiram after detoxification, and most use BQ simultaneously, this study will help inform health care providers to educate people about the aversive reactions of disulfiram and BQ.

## What We Already Know

- Betel quid is an addictive, harmful, and carcinogenic psychotropic substance.
- Consumption of betel quid and alcohol is a rising public health concern in Bhutan and in the Asia-Pacific region.

## What This Article Adds

- Shows the relevance of educating the public about the aversive reactions between Betel Quid and Disulfiram.
- Provides a basis for future research on aversive reactions between Disulfiram and Betel Quid.

## Introduction

Betel nut (BN) is an addictive carcinogenic substance widely consumed across the Asia-Pacific region.^1^ Betel nut is often consumed as Betel quid (BQ) with betel leaf, slaked lime, and tobacco.^1^ Other ingredients like cloves, cardamom, coconut, sugar, and fruit syrups are added to enhance the taste.^2^ Betel nut use is associated with neuronal injury, myocardial infarction, hepatotoxicity, asthma, central obesity, type II diabetes, hyperlipidemia, metabolic syndrome, hypothyroidism, prostate hyperplasia, and infertility.^3^ Half of the oral cancers in Indian subcontinent and in Taiwan were attributed to BQ consumption.^4^

Betel quid is a commonly used and socially and culturally accepted practice in Bhutan. Alcohol consumption is also a very common practice in Bhutan, with the highest prevalence of use in the Southeast Asia Region.^5^ As a result, Bhutan has been recording many deaths from alcohol use and dependence for many years.^6^ People with alcohol use disorders (PwAUDs) experience recurrent or persistent social, interpersonal, personal, academic, or occupational problems.^7^

The Royal Government of Bhutan with the help of the United Nations Office on Drugs and Crime had come up with a long-term medical intervention to treat alcohol dependence. Accordingly, disulfiram, an antabuse (alcohol-abuse deterrent), was introduced in Bhutan in 2016 although it has been used for decades in other countries. Disulfiram is prescribed for individuals opting to stay abstinent from alcohol. It inhibits alcohol metabolism inducing an unpleasant reaction^8^ known as an Alcohol-disulfiram reaction.

Patients admitted to the psychiatric ward at Jigme Dorji Wangchuck National Referral Hospital (JDWNRH) were found to show aversive reactions when Disulfiram was taken with BQ. Similar cases of reactions between disulfiram and BQ were reported in Sri Lanka.^9^ Hence, this study aimed to report the signs and symptoms of aversive reactions between disulfiram and BQ in PwAUDs admitted to JDWNRH.

## Methods

### Study Design and Setting

This prospective observational study was carried out in a 20-bed psychiatry ward of JDWNRH, the only hospital providing specialized psychiatric, detoxification, and counseling services to PwAUDs in Bhutan. Over 12 months, 100 PwAUDs admitted for detoxification, who were on disulfiram, and chewing BQ at the same time, were observed. However, 80 PwAUDs showed an aversive reaction from BQ ingestion alone.

### Data Collection

Disulfiram is usually prescribed and administered on the eighth and ninth day of the detoxification after obtaining consent. For this study, 100 PwAUDs who were administered disulfiram and chewed BQ were observed for a maximum of 48 hours in the ward. A structured questionnaire was used to collect the demographic data and signs and symptoms of an aversive reaction.

### Data Analysis

Data were coded, entered, and double-entered in Epi data version 3.1 and SPSS version 19 was used for analysis. Descriptive statistics such as mean, standard deviation, frequency, and percentage were used to report our findings.

### Ethical Approval

Ethical approval (REBH/Approval/2017/022) was obtained from the Research Ethics Board of Health, Bhutan. Written consent was obtained from the participants. A dedicated room was used for data collection and names of participants were not written on the forms for confidentiality.

## Results

### Demographic Characteristics

Most of the participants were males (81%) with a minimum and maximum age of 24 and 59 years, respectively. The participant’s ages of initiation of alcohol consumption varied considerably across the age groups with a mean age of 22.25 years (SD = 8.47) with 65% of participants initiating alcohol between ages 16 and 30. The mean age of BQ initiation was 23.64 years with 55% of participants initiating BQ between ages 16 and 30 (Table 1).

**Table 1.** Frequency and Percentage of Demographic Characteristics (*N* = 100).

Demographic characteristics	Frequency, *n*	Percentage, *%*
Gender		
Male	81.0	81.0
Female	19.0	19.0
Age (years)		
20-30	14.0	14.0
31-40	50.0	50.0
41-50	26.0	26.0
>51	10.0	10.0
(*M* = 35.51; *SD* = 8.47; *Minimum* = 24; *Maximum* = 59)		
Age of initiation of alcohol (years)		
<15	19.0	19.0
16-30	65.0	65.0
31-45	16.0	16.0
(*M* = 22.25; *SD* = 8.04; *Minimum* = 4; *Maximum* = 43)		
Age of initiation of Betel Nut (years)		
<15	21.0	21.0
16-30	55.0	55.0
31-45	24.0	24.0
(*M* = 23.64; *SD* = 8.65; *Minimum* = 4; *Maximum* = 45)		

Abbreviations: N, total sample; M, mean; SD, standard deviation.

### Observation of Reactions

We included only 20 PwAUDs for observation of aversive reactions between BQ and disulfiram and excluded 80 PwAUDs who showed aversive reactions from BQ ingestion alone. Eighteen PwAUDs developed aversive reactions from disulfiram and BQ presenting with the following signs and symptoms: sweating (50%), diarrhea (35%), dizziness and tremors (20%), palpitations (15%), shortness of breath and nausea (10%), and vomiting and headache (5%) (Table 2). Two PwAUDs showed severe reactions in the form of cardiovascular collapse and had to be transferred to the Emergency Department for resuscitation.

**Table 2.** Signs and Symptoms of Interaction Between Disulfiram and Betel Quid (*N* = 20).

Signs and symptoms	Disulfiram with Betel Quid	Percentage
Sweating	10	50
Diarrhea	7	35
Dizziness	4	20
Tremors	4	20
Palpitations	3	15
Shortness of breath	2	10
Nausea	2	10
Vomiting	1	5
Headache	1	5

N: total number of people with alcohol use disorders (PwAUDs) who were on disulfiram and betel quid.

## Discussion

Betel nut contains arecoline, which is known to stimulate the central nervous system similarly to caffeine and tobacco when used in low doses and cocaine-like action when used in high doses.^7^ Signs and symptoms such as sweating, vomiting, diarrhea, increased saliva, chest pain, abnormal heartbeats, low blood pressure, difficulty in breathing, rapid breathing, seizure, heart attack, coma, and even death observed with BN consumption are attributed to the cholinergic effects of arecoline and the combined effects of betel leaves and lime. This effect depends on the size, weight, health, and the consumer’s patterns of use. The amount of the BQ consumed determines the side effects and is proportional to the amount taken.

This study sought to assess if PwAUDs on disulfiram showed aversive reactions when BQ was taken simultaneously and, if so, what the symptoms could be. Twenty participants in this study had aversive reactions from disulfiram and BQ use. Two of them collapsed with severe cardiovascular symptoms. This indicates the need for educating PwAUDs on safer coping strategies to stay abstinent from alcohol as BQ is used as a substitute for alcohol during detoxification. Most participants in this study initiated using both alcohol and BQ between the ages of 16 and 30. This is well supported by the report on alcohol as a correlate for BQ chewing.^5^ Hence, integrated and targeted awareness sessions may be useful in curbing the initiation of alcohol and BQ use.

Our study was limited by sample size and did not examine the type, frequency, and quantity of BQ consumed. Our findings call for increased education on interactions between disulfiram and BQ, particularly for PwAUDs. Health care providers prescribing disulfiram for alcohol abstinence should be aware of possible aversive reactions in PwAUDs consuming BQ. Our results indicate the potential use of disulfiram for treating BQ addiction, although more rigorous studies are needed to confirm this.

## Conclusion

Our findings have potential implications for preventing BQ addiction, particularly in the Asia-Pacific countries.
